# Targeted proteomics in a population-based study identifies serum PECAM-1 and TRIM21 as inflammation markers for periodontitis

**DOI:** 10.1007/s00784-023-05442-z

**Published:** 2023-12-29

**Authors:** Stefan Lars Reckelkamm, Inga Kamińska, Sebastian-Edgar Baumeister, Mariana Ponce-de-Leon, Benjamin Ehmke, Ewa Rodakowska, Joanna Baginska, Michael Nolde, Karol Adam Kamiński

**Affiliations:** 1 Institute of Health Services Research in Dentistry, University of Münster, Albert-Schweitzer-Campus 1, 48149 Münster, Germany; 2 Clinic for Periodontology and Conservative Dentistry, University of Münster, Münster, Germany; 3 Department of Integrated Dentistry, Medical University of Bialystok, Bialystok, Poland; 4 Chair of Epidemiology at the University Augsburg, University Hospital Augsburg, Augsburg, Germany; 5 Department of Clinical Dentistry-Cariology Section, University of Bergen, 5020 Bergen, Norway; 6 Department of Dentistry Propaedeutics, Medical University of Bialystok, 15-295 Białystok, Poland; 7 Department of Population Medicine and Lifestyle Diseases Prevention, Medical University of Bialystok, Bialystok, Poland

**Keywords:** Proteomics, Inflammation, Periodontitis, Serum markers

## Abstract

**Objectives:**

Periodontitis (PD) can cause systematic inflammation and is associated with various metabolic processes in the body. However, robust serum markers for these relationships are still lacking. This study aims to identify novel circulating inflammation-related proteins associated with PD using targeted proteomics.

**Materials and methods:**

We used population-based, cross-sectional data from 619 participants of the Polish Longitudinal University Study (Bialystok PLUS). Mean pocket probing depth (mPPD) and proportion of bleeding on probing (pBOP) served as exposure variables. Fifty-two inflammation-related proteins were measured using the Olink Target 96 Cardiovascular III and the Olink Target 96 Immune Response panels. Associations between periodontal measures and proteins were tested using covariate-adjusted linear regression models.

**Results:**

At a false discovery rate of < 0.05, we identified associations of mPPD and pBOP with platelet-endothelial cell adhesion molecule-1 (PECAM-1) and tripartite motif–containing protein 21 (TRIM21).

**Conclusion:**

This study revealed novel associations between PD and serum levels of PECAM-1 and TRIM21. Our results suggest that these proteins might be affected by molecular processes that take place in the inflamed periodontium.

**Clinical relevance:**

Novel associations of PECAM-1 and TRIM21 with PD indicate promising serum markers for understanding the disease’s pathophysiological processes and call for further biomedical investigations.

**Supplementary Information:**

The online version contains supplementary material available at 10.1007/s00784-023-05442-z.

## Introduction

Periodontitis (PD), as a complex multifactorial condition, is one of the most common chronic diseases worldwide [1]. Dysbiotic plaque biofilm is the main component initializing the pathological processes. However, extent and progression are largely determined by host-specific features such as an exuberant inflammatory immune response [2]. Previous studies suggest that the relationship between inflammation and PD is in fact mutual with inflammation contributing to periodontal damage and periodontal damage inducing inflammation, a self-sustaining vicious cycle. It is precisely the persistent low-grade inflammation that is suspected to impair overall health [3]. To this date, PD diagnostics focus on assessing the local periodontium, therapies are aimed at managing the biofilm rather than the host’s exacerbating immune system, and the analysis of blood—despite being the most widespread diagnostic procedure in medicine [4]—is rarely used in clinical dental care [5]. This is primarily owed to the lack of appropriate (serum) biomarkers [6].

An appropriate biomarker is a “characteristic that is measured as an indicator of normal biological processes, pathogenic processes, or biological responses to an exposure or intervention” [7]. The concept of using serum proteins to individualize medicine is not new; measurement of C-reactive protein, for instance, has been used to assess inflammation since the protein was discovered nearly 100 years ago [8]. But the potential of available methods has increased dramatically in the last decades [9]. In this regard, “targeted proteomics” describes a rapidly emerging, sensitive, and reliable method to quantify entire protein clusters. The analysis can thus be specifically tailored to a system or tissue under investigation [10]. Recent advances in sequencing technologies provide increasingly powerful tools to identify protein biomarkers, which, in turn, might provide the framework for a variety of future applications: For example, serum biomarkers indicating chronic inflammation in PD could improve diagnostics or help develop and monitor individualized therapies [4].

The ability to generate comprehensive protein profiles at a relatively low cost and the ongoing trend toward individualized, patient-specific medicine drive the increasing adoption of these methods in all medical fields; dental medicine is no exception [11]. So far, however, the application has been limited to studies with small numbers of participants or the investigation of PD in the context of (inflammatory) comorbidities [12–15]. In contrast, this study examined associations between PD measures and circulating levels of 52 inflammation-related serum proteins in a population-based study aiming to identify indicators of systemic PD effects.

## Materials and methods

### Study population

For the Polish Longitudinal University Study (Bialystok PLUS), 3246 individuals aged 20 to 80 were sampled from population registries and invited to participate in a personal interview and medical and dental examinations in 2017–2021 [16]. Proteomic analyses were performed on a randomly selected subsample consisting of 745 participants. After the exclusion of subjects with no dental measurements (*n* = 59) or missing values in covariates (*n* = 67), the analytical sample comprised 619 eligible participants. Relevant ethical approval for the Bialystok PLUS study was granted by the Ethics Committee of the Medical University of Bialystok in conformity with the Declaration of Helsinki (Ethics number: (R-i-002/108/2016), and all participants provided written informed consent.

### Periodontal examination

Periodontal examination was performed by four calibrated dentists under conditions of an epidemiological inquiry (using artificial light but without saliva ejector or air jet) and included pocket probing depth (PPD), gingival recession, and bleeding on probing (BOP). Measurements were performed randomly on either the left- or right-side quadrants and included all erupted teeth except the third molars. Four sites per tooth were assessed; mesiobuccal, distobuccal, midbuccal, and midlingual or midpalatinal. A UNC15 periodontal probe was used for assessment. From these variables, collected as whole millimeters (or binary recorded concerning BOP), the variable mean PPD (mPPD) and proportion of sites with BOP (pBOP) were calculated for each participant. Furthermore, a binary variable indicating gingival health was calculated, according to the epidemiological definition adopted in the proceedings of the 2017 World Workshop on the Classification of Periodontal and Peri-implant Diseases and Conditions, defined as < 10% pBOP with PPD ≤ 3 mm [17].

### Proteomic analysis (OLINK^1^)

Quantification of the protein concentrations in serum was performed using the Olink Target 96 Cardiovascular III and Olink Target 96 Immune Response panel. The approach is based on the proximity extension assay. A detailed description of this method can be found in previous studies [18, 19]. Measured protein concentrations are obtained in a relative quantification unit proposed by Olink, the Normalized Protein eXpression (NPX), which is reported on a log_2_ scale. The increase of one NPX corresponds to a doubling of the protein concentration. Subsequent normalization reduces intra- and inter-assay variability and strengthens the comparability of measurements (both between plates and different studies). Olink provides a classification of measured biomarker proteins based on specific biological processes or disease areas. We combined all proteins of both panels that were assigned to the disease area “inflammatory,” resulting in 74 proteins. The limit of detection (LOD) for each measured protein is further calculated separately for each Olink assay and sample plate. The LOD value is estimated from the negative controls included on every plate, plus three standard deviations, and represents the minimum detectable concentration. The standard deviation is assay-specific and estimated during product validation for every panel. For studies including more than one plate per panel, the maximum observed LOD for each assay is selected as the study LOD. Consequently, all plates included in the study receive the same assay-specific LOD. Proteins for which more than 25% of the samples had values below the LOD were excluded from further analysis. This led to the exclusion of 22 of 74 proteins. For the remaining 52 proteins (a complete list is provided in Supplementary Table 1), values below the LOD were substituted with the respective LOD [19].

### Covariates

Socio-demographic data (i.e., age, sex, school education) as well as self-reported diabetes were obtained from a self-administered questionnaire. For biochemical analyses (including HbA1C), peripheral venous fasting blood was collected in the morning on a visit day, and serum samples were stored at − 80 °C until use. BMI was calculated as weight (in kilograms) divided by the square of height (in meters). Smoking was grouped into never, former, and current smokers. Self-reported alcohol intake was inquired as days on which alcohol was consumed combined with the number of beverage-specific milliliters. The quantity information was used to calculate the amount of alcohol consumed in the last 30 days in grams [20]. For representation purposes in Table 1, the gram per day value is shown. Physical activity was assessed using the International Physical Activity Questionnaire—long format (IPAQ-L) [21]. All IPAQ data was processed using the standardized IPAQ scoring protocol and used as a continuous variable expressed in metabolic equivalent of task (MET) minutes per week. All daily walking, moderate, and vigorous activity time variables were truncated to 3 h, allowing a maximum of 21 h of activity in each category per week. The categorical IPAQ variable was only used to report physical activity in Table 1 and was also built using the IPAQ scoring protocol.

**Table 1:** Study sample characteristics

	Full data	Study sample
*N*	745	619
mPPD (mm)	1.8 (0.7)	1.8 (0.7)
pBOP (%)	15.3 (17.7)	15.0 (17.5)
Age (years)	48.9 (15.2)	47.7 (14.8)
Female	55.7%	54.9%
Diabetes	6.1%	5.0%
HbA1C (%)	5.5 (0.7)	5.5 (0.7)
School education		
≤ 9 years	2.6%	2.1%
= 10–11 years	44.0%	41.4%
≥ 12 years	53.4%	56.5%
Body mass index (kg/m^2^)	27.0 (4.9)	26.8 (4.7)
Smoking status		
Never	41.3%	43.9%
Former	37.4%	36.3%
Current	21.3%	19.7%
Alcohol (g/day)	23.2 (205.6)	22.6 (216.1)
Physical activity per week		
Low	20.8%	20.0%
Medium	45.8%	45.9%
High	33.4%	34.1%

Note: Data presented as mean (SD) or percentages

*mPPD* mean pocket probing depth, *pBOP* proportion of bleeding on probing, *HbA1C* glycosylated hemoglobin type A1C

### Statistical analysis

The associations of pBOP and mPPD with proteins were analyzed using multivariable linear regression models. Each regression model was adjusted for age, sex, smoking, alcohol intake, and physical activity. Continuous covariates were modeled using restricted cubic splines with three knots at fixed quantiles (0.1, 0.5, 0.9) of the distribution [22]. We computed the Benjamini-Hochberg false discovery rate (FDR) on the resulting *p*-values [23]. In a sensitivity analysis, we repeated the primary assessment (by using the adopted definition of gingival health in a third regression model while adjusting the FDR accordingly) to evaluate the relationship between gingival health and proteins. Furthermore, we tested the robustness of our findings in a surrogate analysis by including diabetes and BMI as potential confounders. Analyses were performed using the rms (6.2-0), stats (4.0.5), and ggplot2 (3.3.5) packages in R version 4.0.5 (The R Foundation for Statistical Computing).

## Results

Among the 619 included study participants, the average age was 48 years, 55% were female, and about 1/5 reported current smoking. Periodontal measurements yielded an average of 1.8 mm for the mPPD and a pBOP of 15%. Compared to the full data, the exclusion of individuals with missing values hardly changed the study characteristics with a minimal tendency to a slightly younger and healthier population. The corresponding values (both before and after exclusions) can be found in Table 1.

**Fig. 1:**
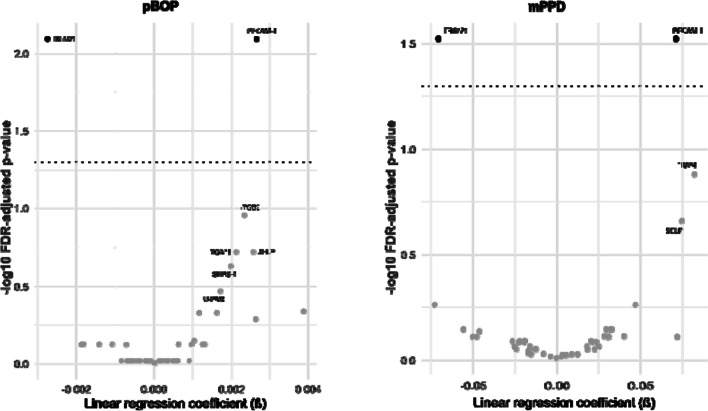
Volcano plots (results from multiple linear regressions) showing results from multiple linear regressions for proportion of bleeding on probing (pBOP) and mean pocket probing depth (mPPD) as exposures; FDR, false discovery rate; dashed line, threshold for FDR adjusted *p*-value = 0.05; protein names are shown when unadjusted *p*-value ≤ 0.05; linear regression models adjusted for age, sex, smoking, alcohol intake, and physical activity; β-coefficients interpretable as change in normalized protein expression (NPX) per unit change in pBOP or mPPD; a list with complete protein names is provided in Supplementary Table 1

**Fig. 2:**
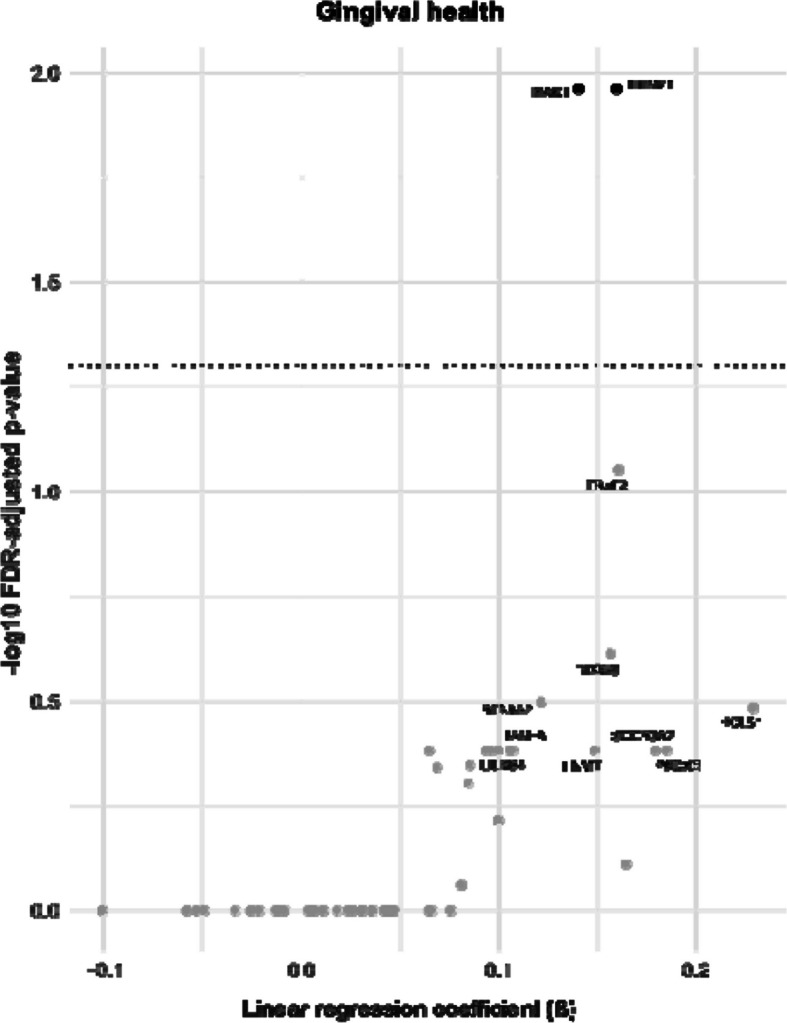
Volcano plot of surrogate analysis (results from multiple linear regressions) showing results from multiple linear regressions for gingival health according to the 2017 World Workshop on the Classification of Periodontal and Peri-implant Diseases and Conditions [17] as exposure; FDR, false discovery rate; dashed line, threshold for FDR adjusted *p*-value = 0.05; protein names are shown if unadjusted *p*-value ≤ 0.05; linear regression models adjusted for age, sex, smoking, alcohol intake, and physical activity; β-coefficients interpretable as the change in normalized protein expression (NPX) when categorized as being a case of gingival health; a list with complete protein names is provided in Supplementary Table 1

Figure 1 presents the FDR adjusted results from multiple linear regressions. At a FDR < 0.05, we found that periodontal measures were associated with two proteins. mPPD and pBOP were positively associated with platelet-endothelial cell adhesion molecule-1 (PECAM-1). In contrast, mPPD and pBOP were inversely associated with tripartite motif–containing protein 21 (TRIM21). FDR adjusted *p*-values for these associations as well as for five additional proteins, that showed associations with *p* < 0.05 without adjustment, are listed in Supplementary Table 2 and 3. The surrogate analysis (Fig. 2) reveals a concordant relation—since in this case, the event is being healthy—between gingival health and TRIM21 as well as a positive link with interleukin-1 receptor-associated kinase 1 (IRAK1). The FDR adjusted *p*-values of this analysis can be found in Supplementary Table 4. Supplementary Table 5 provides the full set of regression results, including regression coefficients, 95% confidence intervals, and both adjusted and unadjusted *p*-values. The surrogate analysis, in which diabetes and BMI were included as additional confounders, mirrored the results of our primary analysis (Supplementary Figure 1).

## Discussion

This explorative study associated 52 serum proteins with two distinct clinical markers for PD, pBOP and mPPD, revealing associations with PECAM-1 and TRIM21. The revealed associations were consistent for both exposures. The association with TRIM21 was also reflected in the surrogate analysis of the epidemiological case definition of gingival health.

**Fig. 3:**
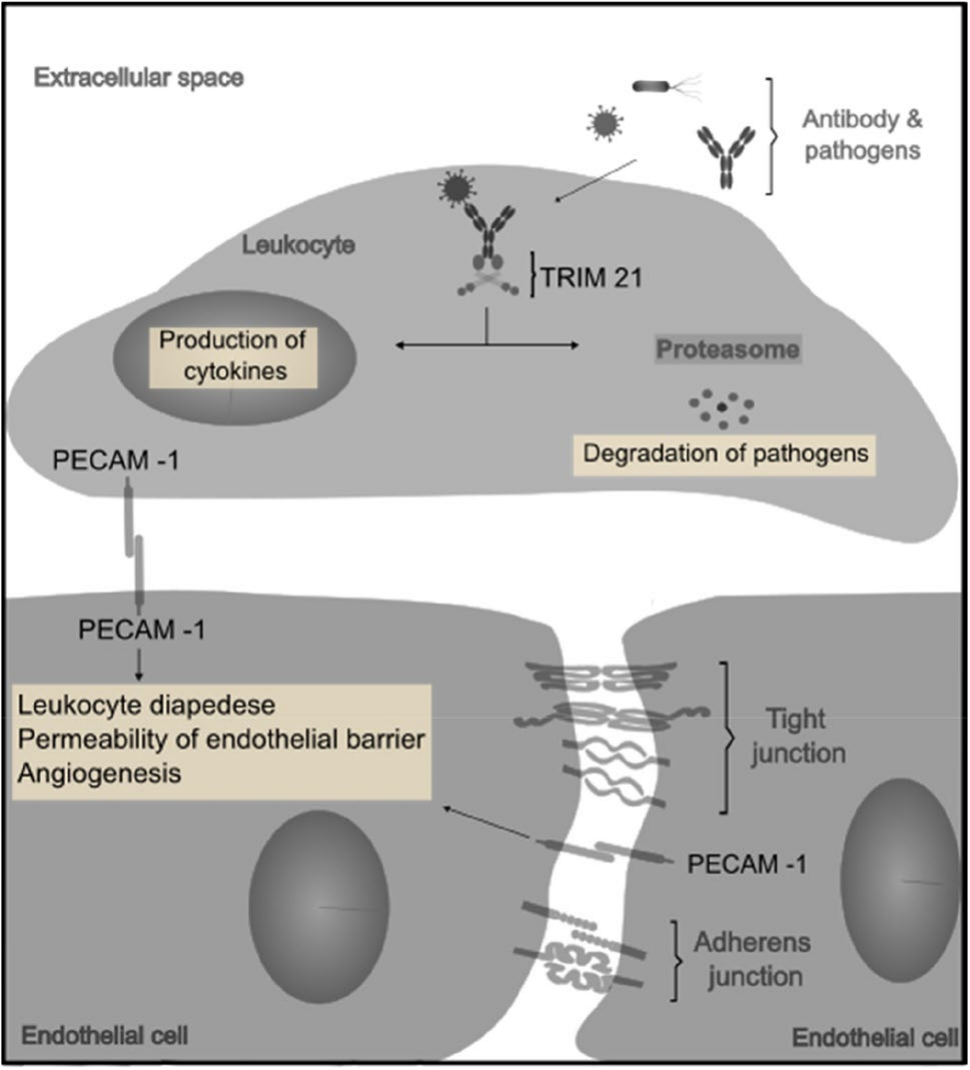
An overview of PECAM-1 and TRIM21. Endothelial cells are, in addition to specialized junctional structures (tight junctions and adherens junctions), connected by cell-specific adhesion molecules, including platelet-endothelial cell adhesion molecule-1 (PECAM-1 also known as CD31). PECAM-1 is both a cellular adhesion and signaling receptor. Platelets and leukocytes also exhibit PECAM-1. Among its functions, this protein is involved in the control of inflammatory and immunological processes. Adhesion of leukocytes to inflamed endothelium initiates cascades that subsequently increase endothelial barrier permeability, promote angiogenesis, and enable leukocyte diapedesis [24, 25]. Tripartite motif–containing protein 21 (TRIM21 or also known as Ro52) is a cytosolic antigen receptor found in most tissues. It binds to pathogens such as viruses or bacteria that enter the cell and, in turn, are attached to antibodies. The resulting complex serves to mark the pathogens for degradation by the proteasome. In addition, TRIM21 activates the NF-κB, AP-1, and IRF signaling pathways, leading to increased production of proinflammatory cytokines [26–28] (illustration based on [27, 29])

PECAM-1 and TRIM21 play multiple roles in basic inflammatory and immunological processes throughout the body (see Fig. 3).

For PECAM-1, we observed a positive association, stating that higher periodontal burden is accompanied by increased serum protein levels. Although the adhesion molecule is well recognized, there is a lack of periodontal research that can be compared with our study. However, a study involving 40 PD patients and 38 healthy controls did reveal a similar relationship in saliva [30]. In contrast to our results, a genetic study tried linking polymorphisms of PECAM-1 with PD by comparing 105 patients and 101 healthy controls with a mean age of 33.3 and 29.1, respectively, but failed to report a significant association [31]. However, for both studies, the limited sample size and, for the latter, the very young average age of the participants must be taken into account. Further evidence corroborating our results is provided by in vitro and animal studies. Two consecutive studies not only demonstrated a link between periodontal infection and PECAM-1 but also described a novel causative local mechanism; *Porphyromonas gingivalis*—a major pathogen of PD—infects endothelial cells and utilizes gingipain proteases to degrade PECAM-1, resulting in a significant local reduction of this molecule and a concomitant impairment of the vascular barrier [32, 33]. A known host response to endothelial damage, and the binding of endothelial cells by leukocytes in general (which combat the pathogens present), is increased gene expression of several chemokines and adhesion molecules, including PECAM-1 [34–36], which presents a possible explanation for the increased serum levels we measured. Elevated serum PECAM-1—contrasting a local reduction—has also been observed in other diseases with a compromised vascular barrier, such as multiple sclerosis [37].

To our knowledge, there are no previous studies investigating a possible linkage between TRIM21 and PD. Nevertheless, TRIM21 is a known autoantibody target (leading to a low serum protein level) in diseases like systemic lupus erythematosus or other rheumatic autoimmune diseases [38], conditions that, in turn, are associated with PD [3, 39]. Additionally, TRIM21 deficiency is associated with an increased proinflammatory cytokine response [40], and animal models show that a defect in the gene region encoding TRIM21 results in a severely impaired immune response, particularly to viruses [38]. Accordingly, TRIM21 deficiency could provide a possible explanation for the high prevalence and copy count of viruses in patients with progressive PD [41]. Future research is essential to examine how this deficit develops. However, it is conceivable that, due to the continuous bacteremia of periodontal pathogens, TRIM21 is degraded to a greater extent in the course of intracellular neutralization, resulting in declined serum levels of this protein (see Fig. 3).

IRAK1, a noteworthy association in the surrogate analysis, is part of a family of serine/threonine kinases essential in the innate immune system. It participates in Toll-like receptor (TLR) and interleukin-1 receptor signaling pathways, which are important in the immune response against pathogens [42]. Imbalances in these pathways have been linked to a variety of illnesses, such as cardiovascular and inflammatory disorders [43]—including periodontal tissue degeneration [44]. Endogenous anti-inflammatory molecules from the IRAK family help regulate the TLR signaling pathway to counter-regulate inflammatory responses. Recent research suggests that the upregulation of IRAK molecules may reduce inflammatory cytokines, but their exact role is still being investigated. However, due to the known drawbacks of using a dichotomized exposure variable, and in light of the fact that the relation could not be established in the analyses of the clinical (metric) characteristics, caution is advised when interpreting this association [45, 46].

Overall, the results of this exploratory research, in combination with previous findings on the biomarkers’ involvement in periodontal and other diseases, provide new insights into the molecular processes surrounding an inflamed periodontium.

Our study has several limitations worth mentioning. First, the study is cross-sectional. That being the case, a reliable temporal separation between exposure and outcome is not possible (risk of reverse causation), and secondly, in observational studies, there is always the risk of systematic bias (e.g., confounding), which may distort the results. We tried to attenuate bias by choosing two correlated but biologically distinct periodontal markers as exposures; pBOP is the hallmark of gingival inflammation and is presumably under greater (and distinct) influence of systemic processes than mPPD, representing the loosening of the periodontal ligament [47]. Thus, the exposures should be at least partially subject to different confounding factors and the consistent associations found for both logically argue in favor of causation. Future research should, however, consider a longitudinal study design. Third, in population data, the estimated prevalence of severe PD is roughly 10%, and the condition is characterized by intermitting episodes of active and passive progression [48]. Hence, the number of participants with a severe and active periodontal burden in our sample is rather small, which might lead to an underestimation of the systemic effects of PD. We, therefore, recommend the evaluation of a more extensive sample and/or a larger number of cases exhibiting acute periodontal lesions. Fourth, participants originated from a single region, which is a potential source of unobserved bias, such as common genetics, and it is unclear whether the results are generalizable. Thus, it is recommended to verify the results in other locations.

## Conclusion

In recent decades, it has become widely accepted that PD is a condition that has not only systemic causes but also systemic effects [3]. However, the biological basis of many observed associations is still unclear, and the direction of the cause-effect relationship is highly controversial. The identification of suitable biomarkers is therefore of great importance. In this context, our study highlights two possible and biologically plausible candidates whose closer investigation may provide new insights into the systemic processes surrounding PD. We suggest further investigation to replicate the retrieved associations of PECAM-1 and TRIM21, if possible, in a longitudinal design with a high number of cases of acute and severe PD.

### Supplementary information

ESM 1 (DOCX 81 kb)

## Data Availability

The Medical University of Bialystok, the implementing body of the Bialystok PLUS study under the direction of Professor Karol A. Kamiński, is the legal owner of all used data. Data from the Bialystok PLUS study are available after data application and the signature of a data transfer agreement with the responsible local authorities.

## Footnotes

1 Olink Proteomics, Uppsala, Sweden
