# Supplementary material for: Targeted proteomics in a population-based study identifies serum PECAM-1 and TRIM21 as inflammation markers for periodontitis
Source: Clin Oral Investig. 2023 Dec 29;28(1):59. doi: 10.1007/s00784-023-05442-z (PMC10756891; doi:10.1007/s00784-023-05442-z)
Supplement: Supplementary file 1 — (DOCX 81 kb) [file 784_2023_5442_MOESM1_ESM.docx]

## Supplementary Table 1 – Analyzed proteins

|  | |
| --- | --- |
| Variable name | Protein name |
| CCL24_OID00592 | C-C motif chemokine 24 |
| FAS_OID00615 | Tumor necrosis factor receptor superfamily member 6 |
| IL-1RT1_OID00613 | Interleukin-1 receptor type 1 |
| IL-1RT2_OID00627 | Interleukin-1 receptor type 2 |
| IL2-RA_OID00570 | Interleukin-2 receptor subunit alpha |
| ITGB2_OID00565 | Integrin beta-2 |
| JAM-A_OID00625 | Junctional adhesion molecule A |
| LTBR_OID00583 | Lymphotoxin-beta receptor |
| MB_OID00616 | Myoglobin |
| MCP-1_OID00576 | Monocyte chemotactic protein 1 |
| MPO_OID00600 | Myeloperoxidase |
| OPG_OID00571 | Osteoprotegerin |
| PECAM-1_OID00652 | Platelet endothelial cell adhesion molecule |
| PON3_OID00642 | Paraoxonase |
| PRTN3_OID00618 | Myeloblastin |
| SCGB3A2_OID00636 | Secretoglobin family 3A member 2 |
| SELP_OID00574 | P-selectin |
| SHPS-1_OID00628 | Tyrosine-protein phosphatase non-receptor type substrate 1 |
| ST2_OID00634 | ST2 protein |
| TFPI_OID00590 | Tissue factor pathway inhibitor |
| TIMP4_OID00585 | Metalloproteinase inhibitor 4 |
| TLT-2_OID00588 | Trem-like transcript 2 protein |
| TNF-R1_OID00649 | Tumor necrosis factor receptor 1 |
| TNF-R2_OID00567 | Tumor necrosis factor receptor 2 |
| TNFSF13B_OID00617 | Tumor necrosis factor ligand superfamily member 13B |
| U-PAR_OID00620 | Urokinase plasminogen activator surface receptor |
| BTN3A2_OID01027 | Butyrophilin subfamily 3 member A2 |
| CCL11_OID00970 | Eotaxin |
| CD83_OID01025 | CD83 antigen |
| CDSN_OID00960 | Corneodesmosin |
| CLEC4A_OID00951 | C-type lectin domain family 4 member A |
| CLEC4C_OID00949 | C-type lectin domain family 4 member C |
| DDX58_OID01018 | Probable ATP-dependent RNA helicase DDX58 |
| FCRL6_OID01006 | Fc receptor-like protein 6 |
| HCLS1_OID00942 | Hematopoietic lineage cell-specific protein |
| HNMT_OID00969 | Histamine N-methyltransferase |
| IFNLR1_OID01010 | Interferon lambda receptor 1 |
| IL10_OID00993 | Interleukin-10 |
| IL12RB1_OID01019 | Interleukin-12 receptor subunit beta-1 |
| IL6_OID00947 | Interleukin-6 |
| IRAK1_OID00950 | Interleukin-1 receptor-associated kinase 1 |
| ITGA11_OID01021 | Integrin alpha-11 |
| KLRD1_OID00995 | Natural killer cells antigen CD94 |
| LILRB4_OID00965 | Leukocyte immunoglobulin-like receptor subfamily B member 4 |
| MGMT_OID00990 | Methylated-DNA-protein-cysteine methyltransferase |
| NTF4_OID00966 | Neurotrophin-4 |
| PRDX5_OID00955 | Peroxiredoxin-5, mitochondrial |
| SH2D1A_OID01002 | SH2 domain-containing protein 1A |
| STC1_OID00999 | Stanniocalcin-1 |
| TPSAB1_OID00941 | Tryptase alpha/beta-1 |
| TRAF2_OID00963 | TNF receptor-associated factor 2 |
| TRIM21_OID00964 | E3 ubiquitin-protein ligase TRIM21 |

## Supplementary Table 2 – Association between mPPD (exposure) and analyzed proteins (outcome)

| **Protein** | **FDR-adjusted p-value** |
| --- | --- |
| PECAM-1 | 0.030 |
| TRIM 21 | 0.030 |
| TIMP4 | 0.131 |
| SELP | 0.219 |

**Supp. Table 2** showing FDR-adjusted p-values from multiple linear regressions models adjusted for age, sex, smoking, alcohol intake, and physical activity when unadjusted p-value ≤ 0.05

Abbreviations: mPPD, mean pocket probing depth, FDR, False discovery rate.

## Supplementary Table 3 – Association between pBOP (exposure) and analyzed proteins (outcome)

| **Protein** | **FDR-adjusted p-value** |
| --- | --- |
| PECAM-1 | 0.008 |
| TRIM21 | 0.008 |
| ITGB2 | 0.111 |
| SELP | 0.190 |
| ITGA11 | 0.190 |
| SHPS-1 | 0.234 |
| U-PAR | 0.339 |

**Supp. Table 3** showing FDR-adjusted p-values from multiple linear regressions models adjusted for age, sex, smoking, alcohol intake, and physical activity when unadjusted p-value ≤ 0.05

Abbreviations: pBOP, proportion of bleeding on probing, FDR, False discovery rate.

## Supplementary Table 4 – Association between gingival health (exposure) and analyzed proteins (outcome)

| **Protein** | **FDR-adjusted p-value** |
| --- | --- |
| TRIM21 | 0.011 |
| IRAK1 | 0.011 |
| TRAF2 | 0.089 |
| DDX58 | 0.244 |
| BTN3A2 | 0.317 |
| HCLS1  PRDX5  JAM-A  SCGB3A2  LILRB4  HNMT | 0.414  0.414  0.414  0.414  0.414  0.414 |

**Supp. Table 4** showing FDR-adjusted p-values from multiple linear regressions models adjusted for age, sex, smoking, alcohol intake, and physical activity when unadjusted p-value ≤ 0.05

Abbreviations: FDR, False discovery rate.

**Supplementary Table 5 – Regression Analyses Results: Association between periodontal parameters (exposure) and analyzed proteins (outcome)**

|  | **mPPD** | | | **pBOP** | | | **Gingival health** | | |
| --- | --- | --- | --- | --- | --- | --- | --- | --- | --- |
| Protein | Beta | P | pFDR | Beta | P | pFDR | Beta | P | pFDR |
| PECAM_1_OID00652 | 0.071  [0.03-0.112] | 7.47e-04 | 0.030 | 0.003  [0.001-0.004] | 3.10e-04 | 0.010 | -0.013 [-0.092-0.066] | 0.750 | 1.000 |
| TRIM21_OID00964 | -0.071 [-0.114--0.028] | 1.15e-03 | 0.030 | -0.003 [-0.004--0.001] | 2.78e-04 | 0.010 | 0.16 [0.079-0.241] | 1.19e-04 | 0.010 |
| TIMP4_OID00585 | 0.082 [0.022-0.142] | 0.010 | 0.130 | 0 [-0.002-0.002] | 0.870 | 0.960 | -0.034 [-0.149-0.081] | 0.560 | 1.000 |
| SELP_OID00574 | 0.074 [0.013-0.135] | 0.020 | 0.220 | 0.003 [0-0.005] | 0.020 | 0.190 | 0.081 [-0.036-0.197] | 0.180 | 0.870 |
| U_PAR_OID00620 | 0.047 [-0.001-0.095] | 0.060 | 0.550 | 0.002 [0-0.003] | 0.050 | 0.340 | 0.046 [-0.046-0.138] | 0.330 | 1.000 |
| HNMT_OID00969 | -0.073 [-0.15-0.004] | 0.060 | 0.550 | -0.001 [-0.004-0.002] | 0.540 | 0.960 | 0.148 [0.002-0.295] | 0.050 | 0.410 |
| TNFSF13B_OID00617 | 0.029 [-0.009-0.067] | 0.130 | 0.720 | 0.001 [0-0.002] | 0.090 | 0.470 | 0.007 [-0.066-0.08] | 0.860 | 1.000 |
| TNF_R1_OID00649 | 0.032 [-0.01-0.073] | 0.140 | 0.720 | 0.001 [0-0.002] | 0.160 | 0.710 | -0.022 [-0.102-0.058] | 0.590 | 1.000 |
| MB_OID00616 | -0.056 [-0.123-0.011] | 0.100 | 0.720 | -0.001 [-0.003-0.002] | 0.600 | 0.960 | 0.065 [-0.063-0.193] | 0.320 | 1.000 |
| SH2D1A_OID01002 | 0.033 [-0.009-0.075] | 0.130 | 0.720 | 0 [-0.002-0.001] | 0.920 | 0.960 | 0.026 [-0.054-0.106] | 0.530 | 1.000 |
| CDSN_OID00960 | -0.047 [-0.111-0.018] | 0.150 | 0.730 | -0.001 [-0.004-0.001] | 0.210 | 0.750 | 0.099 [-0.023-0.221] | 0.110 | 0.610 |
| TNF_R2_OID00567 | 0.04 [-0.018-0.098] | 0.180 | 0.770 | 0.001 [-0.001-0.003] | 0.230 | 0.750 | 0.011 [-0.101-0.122] | 0.850 | 1.000 |
| OPG_OID00571 | 0.028 [-0.014-0.071] | 0.190 | 0.770 | 0 [-0.001-0.002] | 0.520 | 0.960 | 0.024 [-0.057-0.105] | 0.570 | 1.000 |
| CCL24_OID00592 | 0.072 [-0.049-0.192] | 0.250 | 0.780 | 0.004 [0-0.008] | 0.070 | 0.460 | -0.059 [-0.29-0.173] | 0.620 | 1.000 |
| TLT_2_OID00588 | 0.031 [-0.018-0.08] | 0.220 | 0.780 | 0.001 [-0.001-0.003] | 0.270 | 0.750 | 0.043 [-0.051-0.137] | 0.370 | 1.000 |
| SHPS_1_OID00628 | 0.031 [-0.019-0.081] | 0.230 | 0.780 | 0.002 [0-0.004] | 0.030 | 0.230 | 0.084 [-0.012-0.18] | 0.090 | 0.500 |
| IL10_OID00993 | -0.048 [-0.133-0.037] | 0.270 | 0.780 | 0 [-0.003-0.003] | 0.930 | 0.960 | -0.054 [-0.216-0.109] | 0.520 | 1.000 |
| PRDX5_OID00955 | -0.05 [-0.139-0.038] | 0.270 | 0.780 | -0.002 [-0.005-0.001] | 0.230 | 0.750 | 0.185 [0.016-0.353] | 0.030 | 0.410 |
| IL_1RT2_OID00627 | -0.02 [-0.06-0.021] | 0.340 | 0.810 | 0 [-0.002-0.001] | 0.860 | 0.960 | 0.036 [-0.042-0.114] | 0.360 | 1.000 |
| FAS_OID00615 | -0.022 [-0.071-0.026] | 0.360 | 0.810 | 0 [-0.002-0.001] | 0.680 | 0.960 | 0.085 [-0.007-0.177] | 0.070 | 0.450 |
| IL12RB1_OID01019 | -0.019 [-0.059-0.02] | 0.340 | 0.810 | -0.001 [-0.002-0.001] | 0.300 | 0.750 | 0.068 [-0.007-0.144] | 0.070 | 0.460 |
| BTN3A2_OID01027 | -0.027 [-0.078-0.025] | 0.310 | 0.810 | -0.001 [-0.002-0.001] | 0.460 | 0.960 | 0.121 [0.023-0.219] | 0.020 | 0.320 |
| CD83_OID01025 | 0.02 [-0.023-0.063] | 0.360 | 0.810 | 0.001 [-0.001-0.002] | 0.500 | 0.960 | 0.044 [-0.038-0.126] | 0.300 | 1.000 |
| IL2_RA_OID00570 | 0.021 [-0.031-0.074] | 0.430 | 0.820 | 0 [-0.002-0.002] | 0.870 | 0.960 | 0.044 [-0.057-0.145] | 0.390 | 1.000 |
| TFPI_OID00590 | -0.02 [-0.065-0.025] | 0.390 | 0.820 | 0 [-0.002-0.001] | 0.810 | 0.960 | 0.045 [-0.041-0.131] | 0.300 | 1.000 |
| CCL11_OID00970 | 0.024 [-0.031-0.078] | 0.390 | 0.820 | 0 [-0.002-0.002] | 0.750 | 0.960 | 0.099 [-0.004-0.202] | 0.060 | 0.410 |
| TRAF2_OID00963 | -0.023 [-0.078-0.032] | 0.410 | 0.820 | -0.001 [-0.003-0.001] | 0.270 | 0.750 | 0.161 [0.057-0.265] | 0.000 | 0.090 |
| MPO_OID00600 | -0.026 [-0.095-0.043] | 0.470 | 0.860 | 0.001 [-0.001-0.004] | 0.290 | 0.750 | 0.076 [-0.056-0.207] | 0.260 | 1.000 |
| KLRD1_OID00995 | 0.025 [-0.045-0.095] | 0.480 | 0.860 | 0 [-0.002-0.003] | 0.770 | 0.960 | -0.009 [-0.142-0.124] | 0.890 | 1.000 |
| LILRB4_OID00965 | 0.018 [-0.036-0.072] | 0.510 | 0.860 | 0 [-0.002-0.002] | 0.830 | 0.960 | 0.105 [0.003-0.208] | 0.040 | 0.410 |
| CLEC4A_OID00951 | -0.016 [-0.064-0.031] | 0.500 | 0.860 | 0.001 [-0.001-0.002] | 0.470 | 0.960 | -0.011 [-0.102-0.08] | 0.810 | 1.000 |
| MCP_1_OID00576 | -0.013 [-0.064-0.038] | 0.620 | 0.890 | 0 [-0.002-0.002] | 0.880 | 0.960 | 0.096 [-0.002-0.193] | 0.050 | 0.410 |
| PON3_OID00642 | -0.025 [-0.106-0.057] | 0.550 | 0.890 | -0.001 [-0.004-0.002] | 0.630 | 0.960 | 0.004 [-0.152-0.159] | 0.960 | 1.000 |
| DDX58_OID01018 | -0.017 [-0.079-0.046] | 0.600 | 0.890 | -0.001 [-0.003-0.002] | 0.610 | 0.960 | 0.157 [0.039-0.274] | 0.010 | 0.240 |
| IL6_OID00947 | 0.023 [-0.063-0.108] | 0.610 | 0.890 | 0 [-0.003-0.003] | 0.880 | 0.960 | -0.101 [-0.264-0.062] | 0.220 | 1.000 |
| NTF4_OID00966 | 0.018 [-0.056-0.093] | 0.630 | 0.890 | -0.001 [-0.003-0.002] | 0.700 | 0.960 | 0.005 [-0.137-0.148] | 0.940 | 1.000 |
| IFNLR1_OID01010 | -0.013 [-0.063-0.038] | 0.620 | 0.890 | 0 [-0.002-0.001] | 0.640 | 0.960 | 0.093 [-0.003-0.189] | 0.060 | 0.410 |
| CLEC4C_OID00949 | -0.018 [-0.096-0.061] | 0.660 | 0.910 | -0.001 [-0.003-0.002] | 0.670 | 0.960 | 0.031 [-0.12-0.181] | 0.690 | 1.000 |
| SCGB3A2_OID00636 | -0.018 [-0.108-0.073] | 0.700 | 0.920 | -0.002 [-0.005-0.001] | 0.260 | 0.750 | 0.179 [0.007-0.351] | 0.040 | 0.410 |
| TPSAB1_OID00941 | -0.017 [-0.108-0.074] | 0.710 | 0.920 | 0.001 [-0.003-0.004] | 0.710 | 0.960 | 0.064 [-0.109-0.237] | 0.470 | 1.000 |
| ITGA11_OID01021 | -0.008 [-0.058-0.041] | 0.730 | 0.930 | 0.002 [0-0.004] | 0.010 | 0.190 | 0.03 [-0.064-0.124] | 0.530 | 1.000 |
| PRTN3_OID00618 | 0.012 [-0.08-0.105] | 0.790 | 0.940 | 0.003 [-0.001-0.006] | 0.110 | 0.520 | 0.047 [-0.129-0.223] | 0.600 | 1.000 |
| ST2_OID00634 | 0.009 [-0.052-0.07] | 0.780 | 0.940 | 0.001 [-0.002-0.003] | 0.640 | 0.960 | -0.05 [-0.166-0.067] | 0.410 | 1.000 |
| HCLS1_OID00942 | -0.016 [-0.116-0.085] | 0.760 | 0.940 | 0 [-0.003-0.004] | 0.990 | 0.990 | 0.229 [0.038-0.42] | 0.020 | 0.330 |
| LTBR_OID00583 | 0.003 [-0.032-0.038] | 0.870 | 0.940 | 0 [-0.001-0.002] | 0.580 | 0.960 | 0.064 [-0.002-0.131] | 0.060 | 0.410 |
| ITGB2_OID00565 | 0.003 [-0.045-0.052] | 0.890 | 0.940 | 0.002 [0.001-0.004] | 0.010 | 0.110 | 0.042 [-0.05-0.134] | 0.370 | 1.000 |
| IRAK1_OID00950 | 0.003 [-0.036-0.042] | 0.870 | 0.940 | 0 [-0.002-0.001] | 0.760 | 0.960 | 0.14 [0.067-0.214] | 2.11e-04 | 0.010 |
| STC1_OID00999 | 0.005 [-0.049-0.059] | 0.870 | 0.940 | 0.002 [0-0.003] | 0.090 | 0.470 | 0.036 [-0.067-0.138] | 0.500 | 1.000 |
| FCRL6_OID01006 | 0.006 [-0.073-0.084] | 0.890 | 0.940 | 0.001 [-0.002-0.004] | 0.510 | 0.960 | -0.026 [-0.176-0.124] | 0.730 | 1.000 |
| JAM_A_OID00625 | 0.002 [-0.05-0.055] | 0.930 | 0.960 | 0 [-0.002-0.002] | 0.990 | 0.990 | 0.107 [0.007-0.207] | 0.040 | 0.410 |
| MGMT_OID00990 | -0.004 [-0.122-0.113] | 0.940 | 0.960 | -0.001 [-0.005-0.003] | 0.740 | 0.960 | 0.164 [-0.059-0.388] | 0.150 | 0.780 |
| IL_1RT1_OID00613 | 0 [-0.033-0.033] | 0.980 | 0.980 | 0.001 [-0.001-0.002] | 0.280 | 0.750 | 0.018 [-0.045-0.081] | 0.580 | 1.000 |

**Supp. Table 5** showing the regression output for multiple linear regressions models adjusted for age, sex, smoking, alcohol intake, and physical activity.

Abbreviations: mPPD, mean pocket probing depth; pBOP, proportion of bleeding on probing; Beta, Linear regression coefficient (β); P, P-value; pFDR, P-Value adjusted by false discovery rate

**Supplementary Figure 1: Volcano plots for confounder extended regressions**


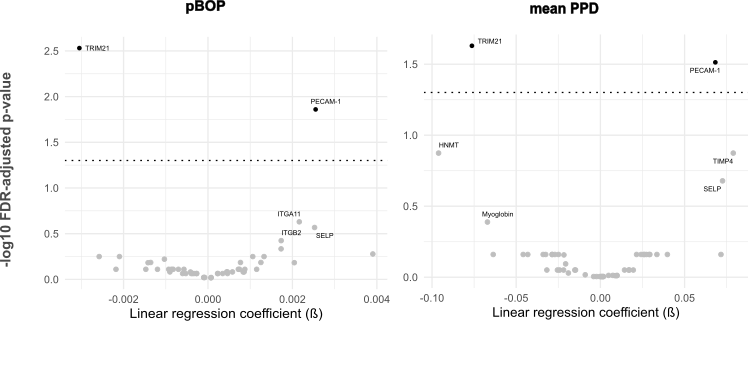


**Supp. Figure 1 Volcano plot** showing results from multiple linear regressions for proportion of bleeding on probing (pBOP) and mean pocket probing depth (mean PPD) as exposures; FDR, false discovery rate; Dashed line, threshold for FDR adjusted p-value = 0.05; Protein names are shown if unadjusted p-value <= 0.05; Linear regression models adjusted for age, sex, smoking, alcohol intake, physical activity, bmi and diabetes; β-Coefficients interpretable as change in normalized protein expression (NPX) per unit change in pBOP or mean PPD
